# Laparoscopic distal pancreatectomy for metastatic melanoma originating from the choroidal membrane: a case report

**DOI:** 10.1186/s40792-021-01345-x

**Published:** 2021-12-20

**Authors:** Shigeaki Baba, Yuji Akiyama, Fumitaka Endo, Haruka Nikai, Ryo Sugimoto, Akira Umemura, Hirokatsu Katagiri, Yasushi Hasegawa, Takeshi Iwaya, Hiroyuki Nitta, Keisuke Koeda, Tamotsu Sugai, Akira Sasaki

**Affiliations:** 1 Department of Surgery, Iwate Medical University School of Medicine, Iwate, Japan; 2 Department of Molecular Diagnostic Pathology, Iwate Medical University School of Medicine, Iwate, Japan; 3 Department of Surgery, Keio University School of Medicine, Tokyo, Japan; 4 Department of Medical Safety Science, Iwate Medical University School of Medicine, Iwate, Japan

**Keywords:** Malignant melanoma, Choroidal, Metastatic pancreatic tumor, Adjuvant molecular-targeted chemotherapy

## Abstract

**Background:**

Metastatic melanoma originating from the choroidal membrane is extremely rare. Here, we report a case of laparoscopic distal pancreatectomy for malignant melanoma that developed after heavy-particle therapy for malignant choroidal melanoma.

**Case presentation:**

A 43-year-old Japanese woman underwent 70 Gy heavy-particle radiotherapy for a right choroidal malignant melanoma. Positron emission tomography-computed tomography examination was performed 4 years after treatment, when contrast accumulation was observed on the posterior wall of the stomach. Endoscopic ultrasonography and computed tomography showed a mass with contrast enhancement in contact with the stomach wall. Based on the imaging findings, a gastrointestinal stromal tumor of the posterior wall of the lower gastric corpus with extramural growth was suspected. Laparoscopic surgery was performed under general anesthesia. A black-pigmented tumor originating from the pancreas was discovered. Following an intraoperative diagnosis of metastasis of malignant melanoma, a laparoscopic distal pancreatectomy was performed. The pathological diagnosis was pancreatic metastasis of malignant melanoma. The patient was treated with adjuvant immune checkpoint inhibitors and chemotherapy after surgery, which led to long-term survival.

**Conclusions:**

Including this case, only eight case reports on pancreatic resection for metastatic ocular malignant melanoma have been reported. The ocular malignant melanoma with distant metastasis has a poor prognosis. Therefore, in our case, careful follow-up is required. A single pancreatic metastasis from a malignant melanoma of the choroid can be successfully managed by laparoscopic radical resection of the pancreas, and molecularly targeted adjuvant chemotherapy.

## Background

Malignant melanoma affects the skin and mucous membranes and has a very poor prognosis. It can metastasize to any part of the body from an early stage. The skin, lymph nodes, lungs, liver, brain, and bones are the organs that frequently experience metastasis [1, 2]. Metastases are infrequent in the gastrointestinal tract (1–7%) [1, 3], and pancreatic metastases are extremely rare (< 1%) [2]. Here, we report a case of laparoscopic resection of the pancreatic tail for malignant melanoma that developed after heavy-ion radiotherapy for malignant choroidal melanoma.

## Case presentation

**Fig. 1:**
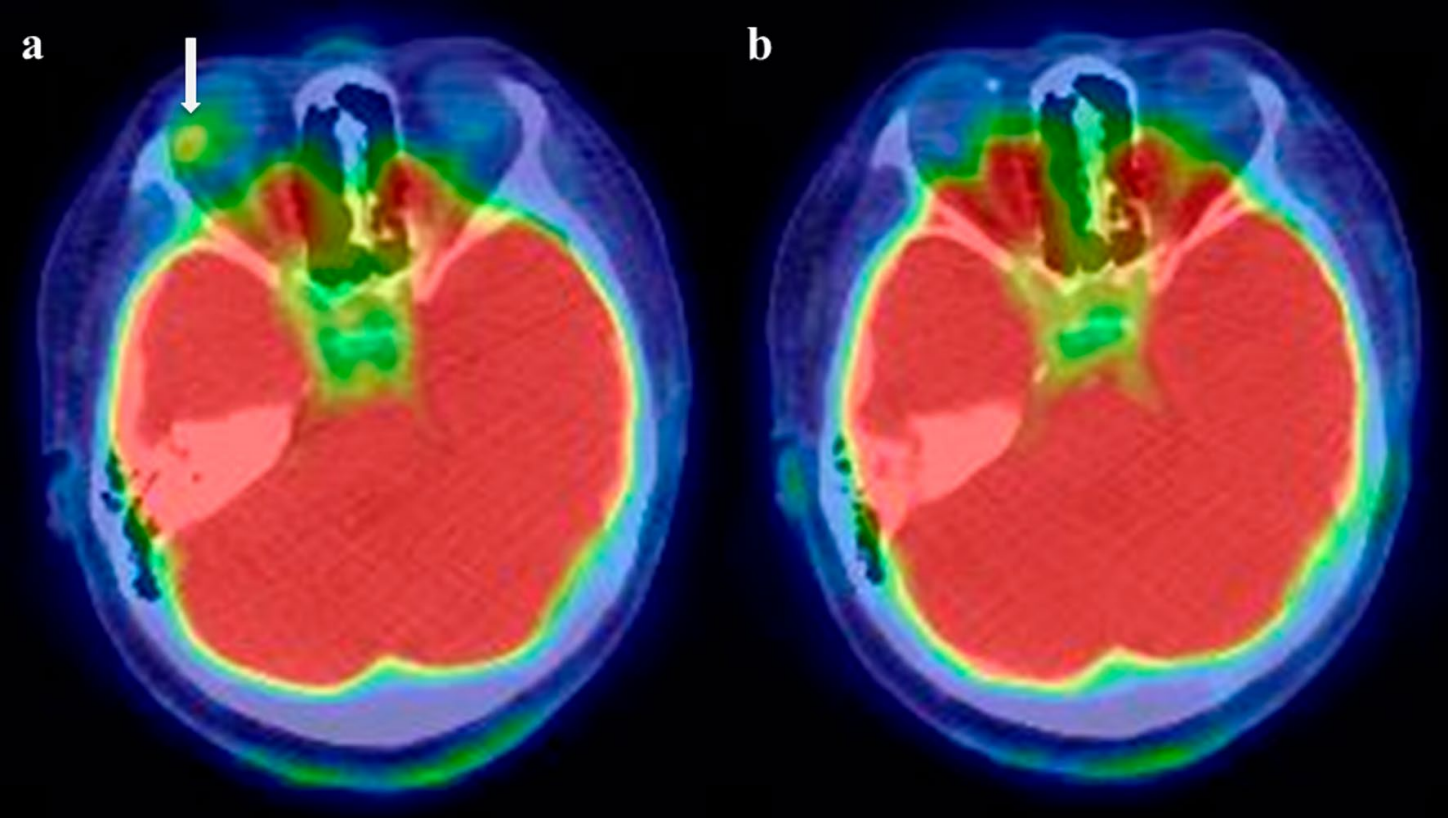
Pre-treatment positron emission tomography (PET) showed significant fluorodeoxyglucose (FDG) accumulation in the right eye (**a**). PET after heavy-particle radiotherapy showed no FDG accumulation in the lesion (**b**)

**Fig. 2:**
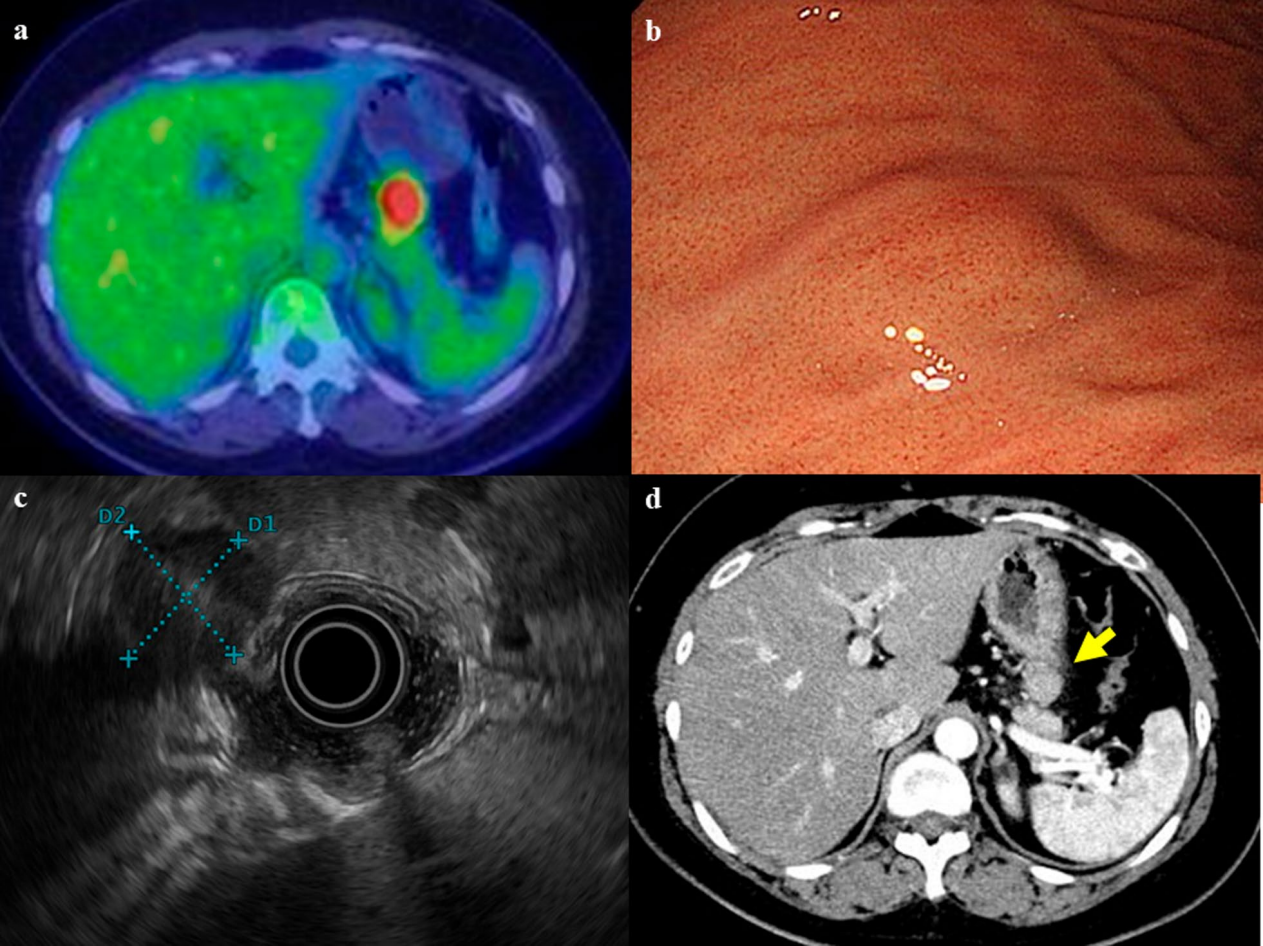
Positron emission tomography (PET) showed significant fluorodeoxyglucose accumulation (FDG) with a maximum standardized uptake value of 10.9 (**a**). Gastrointestinal endoscopic examination showed a smooth and protrusive lesion at the great curvature wall of the gastric body (**b**). Endoscopic ultrasound showed a hypoechoic exogenous mass and size of about 2.2 × 2.0 cm in the post wall of the stomach (**c**). Preoperative abdominal computed tomography scan showed an occupying lesion in the gastric wall (**d**). The arrow indicates the continuous mass on the stomach wall

The patient was a 43-year-old Japanese woman who underwent 70 Gy heavy-particle radiotherapy administered as 5 fractions of 14 Gy for a right choroidal malignant melanoma (T3a, N0, M0 clinical-stage IIB according to the 8th edition of the International Union Against Cancer classification) (Fig. 1a). Positron emission tomography-computed tomography (PET-CT) performed post-treatment revealed no abnormal accumulation of 2-deoxy-2-(fluorine-18) fluoro-d-glucose, and the complete response was recorded (Fig. 1b). PET-CT examination was subsequently performed regularly every 6 months, and no recurrence was noted up to 4 years after treatment, when contrast accumulation (maximum standardized uptake value 10.9) was observed on the posterior wall of the stomach (Fig. 2a).

Gastrointestinal endoscopic examination revealed a smooth, protruding lesion on the wall of the greater curvature of the stomach (Fig. 2b). Endoscopic ultrasonography (EUS) revealed extramural growth of a hypoechoic tumor with the presence of blood flow inside, on the posterior wall of the stomach that was approximately 2.2 × 2.0 cm in size (Fig. 2c). CT showed a mass with contrast enhancement in contact with the stomach wall (Fig. 2d). Based on the imaging findings, a gastrointestinal stromal tumor (GIST) at the posterior wall of the lower gastric corpus with extramural growth was suspected. Therefore, the patient was referred to our hospital for treatment.

**Fig. 3:**
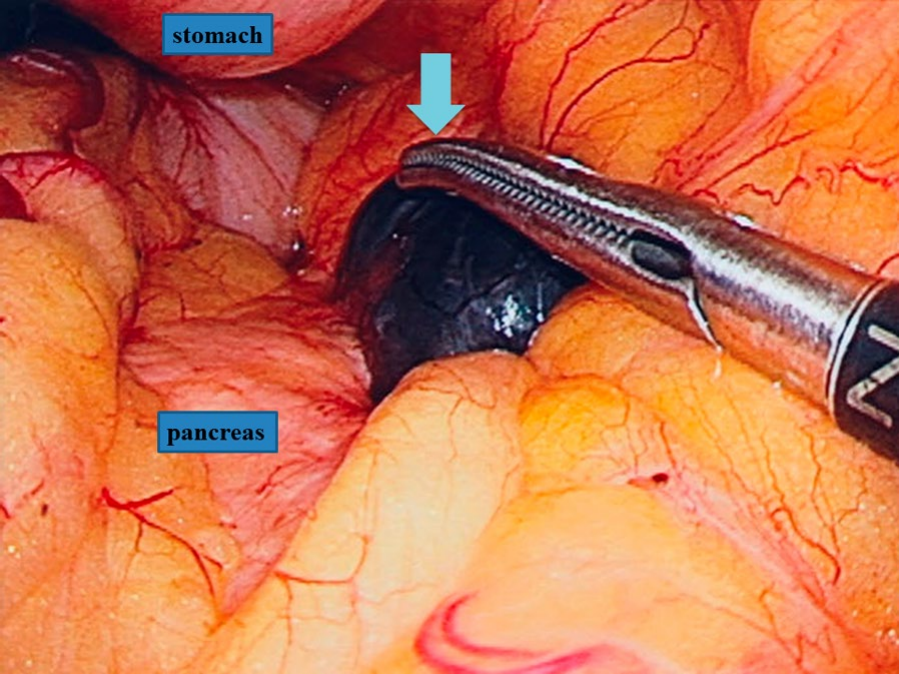
Black color mass (arrow) in the pancreas was found during the laparoscopic operation

Laparoscopic surgery was performed under general anesthesia. A black-pigmented tumor originating from the body of the pancreas was discovered to be in contact with the posterior wall of the stomach (Fig. 3), but continuity was not observed. Based on the intraoperative findings, metastasis of the malignant choroidal melanoma to the pancreas was diagnosed. Since no other lesions were discovered in the abdominal cavity, it was determined that resection would be possible, and laparoscopic pancreatic tail resection was performed.

**Fig. 4:**
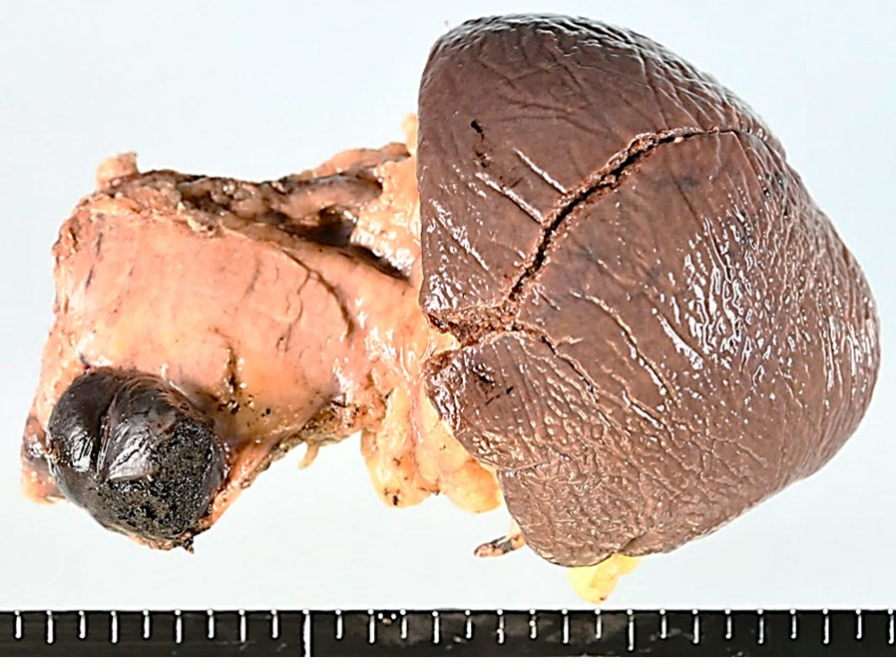
Resected pancreas and spleen with single mass formation of a highly pigmented nodular lesion. The maximum tumor diameter was 27 mm

**Fig. 5:**
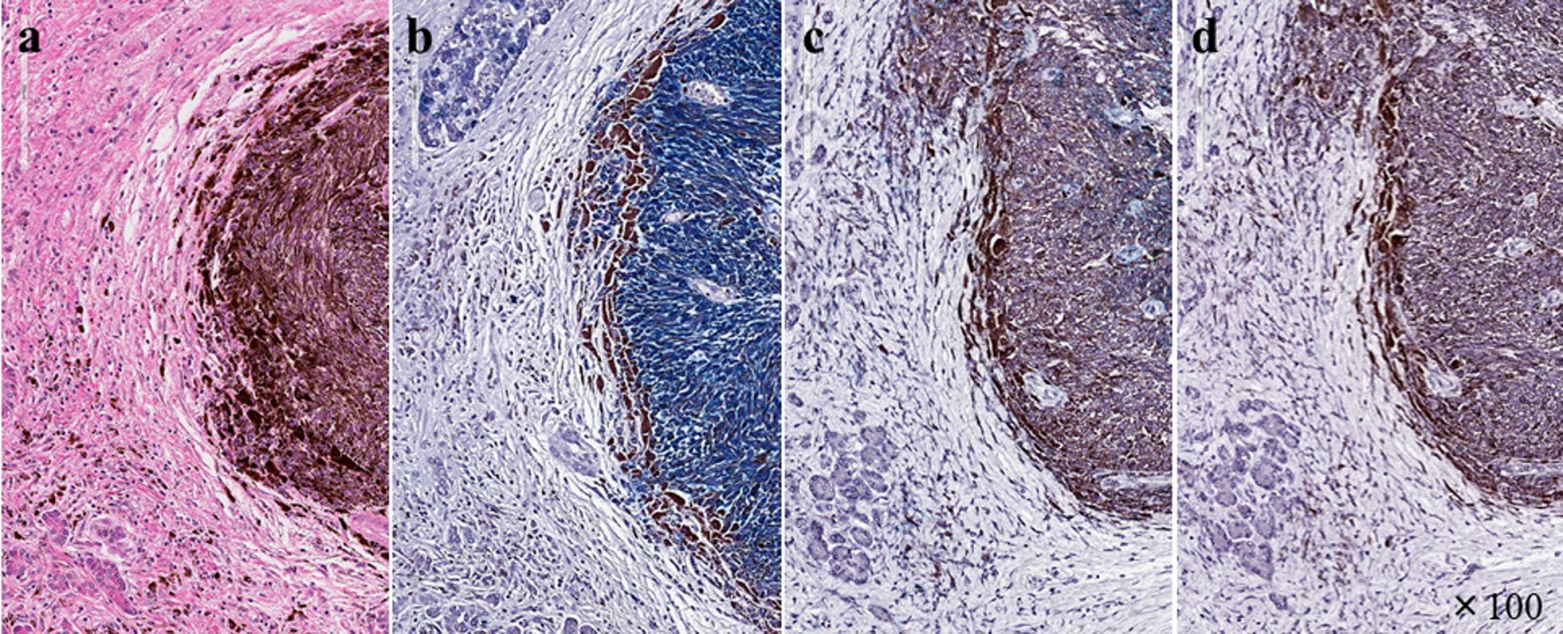
Surgically resected specimen of a pancreatic mass. Original magnification × 100. **a** Hematoxylin and eosin staining, **b** S100 protein, **c** HMB-45, **d** Melan A. Spindle cell appearance with melanin pigment production showed diffuse proliferation. **a** Immunohistochemical experiments showed positive signals for S100 protein (**b**), HNB-45 (**c**), and Melan A (**d**)

The resected tumor comprised a single black nodule with a long-axis diameter of 27 mm (Fig. 4). Hematoxylin and eosin staining revealed spindle cells with melanin pigment production (Fig. 5a). Immunohistological examination showed positive signals for the S100 protein, HNB-45, and Melan A, confirming the diagnosis (Fig. 5b–d). On postoperative day 20, a grade IIIa pancreatic fistula (Clavien–Dindo classification version 2) developed, but amelioration was achieved using conservative treatment. The patient was discharged on postoperative day 50.

Administration of 3 mg/kg nivolumab was initiated as adjuvant chemotherapy 2 months after surgery, but 2 months later multiple lung metastases were detected by CT. Therefore, administration of 3 mg/kg ipilimumab was initiated. Following the first dose, the patient developed colitis as an immune-related adverse event and grade 3 diarrhea (based on the Common Terminology Criteria for Adverse Events version 5.0 classification), the latter of which was ameliorated by oral administration of 30 mg prednisolone. There was an outbreak of dermatitis, which was also judged to be an immune-related adverse event, and the administration of ipilimumab was discontinued. Two courses of 800 mg/m^2^ dacarbazine were administered 12 months postoperatively, but the treatment was discontinued due to metastasis to the left kidney. Nivolumab administration resumed 28 months after surgery. The patient was alive after 17 courses of nivolumab without symptoms or exacerbation of the lesion.

## Discussion

Malignant melanoma normally occurs in sun-exposed skin, but onset may occasionally occur in the eyes, nervous system, nose, mouth, digestive system, or internal organs [4]. Ocular malignant melanoma occurs in all age groups but is frequently seen in people aged 65 years and older [5, 6]. It accounts for only 3–5% of malignant melanomas (annual incidence of approximately 1.6 per million) and is commonly uveal [7]. Uveal melanomas include choroidal, ciliary body, and iris melanomas, of which 90% [4] have a fatal prognosis [8]. Malignant melanoma of the uvea arises from melanocytes in the uveal tract. Since there are no lymphatic vessels in the eye, melanoma spreads hematogenously [9]. Metastasis in any region of the body occurs in 40‒50% of cases [10]. In approximately half of the patients with distant metastases, the internal organs are affected, with the highest frequency (approximately 95%) being in the liver [9]. The median survival time for liver metastasis is 6‒7 months, and the estimated 1-year survival is 10‒15% [8, 11, 12].

Metastatic pancreatic tumors are rare, with a reported frequency of 2% of pancreatic tumors [13–16]. In a study of 819 autopsy cases by Hruban et al. metastatic pancreatic tumors originated from the lung in 25% of cases, the breast in 13%, and malignant melanoma in 11% [17]. In 190 patients treated surgically, the primary organs were lung, kidney, and malignant melanoma, with 23%, 15%, and 5% of the cases, respectively [17].

In a search of PubMed for reports from 1966 to May 2021 with the keywords "ocular melanoma," "metastasis," and "pancreas," we identified 46 reports of resected melanoma with pancreatic metastasis [18], of which 7 were metastases from ocular primary tumors [19–24]. The primary sites of occurrence without ocular involvement were as follows: skin, 14 cases; nasal cavity, 2 cases; oral cavity, 1 case; unknown, 6 cases; and no indication, 16 cases. The mean age at recurrence was 51.3 years, and the median time to recurrence was 4.0 years (range 0.5–28.0 years). The median tumor size was 4.8 cm (range 1.3–18.0 cm). Twenty (43%) patients died after pancreatic tumor resection, with a median postoperative survival of 10.0 months (range 3.0–26.0 months).

Among the 8 reported cases of pancreatic metastasis from ocular malignant melanoma, including this case, 6 (75%) patients were women and 2 (25%) were men; the mean age was 55.4 years. Preoperative diagnosis by CT was performed in six (75%) patients using magnetic resonance imaging, and PET-CT was performed during additional examinations. Endoscopic ultrasound-guided fine-needle aspiration was used for diagnosis in some cases. The median time from initial resection to the onset of pancreatic metastasis was 12.0 years (range 5.0–28.0 years), and the median tumor size was 3.5 cm (range 2.2–18.0 cm). Distal pancreatectomy with simultaneous splenectomy was performed in four patients, pancreaticoduodenectomy in two, pylorus-preserving pancreaticoduodenectomy in one, and total pancreatectomy in one. The median follow-up period after pancreatic resection was 15.5 months (range 6.0–40.0 months) and there were no reported deaths (Table 1). The findings in these reports suggest that the recurrence period after ocular malignant melanoma might be longer than that of pancreatic metastases originating from other organs. The 5-year survival rate for distant metastases from ocular malignant melanoma to any site is 39% when complete resection is performed [25], but there have been no cases of survival when resection is not performed. Therefore, complete resection seems to prolong survival [20,25‒28]. Reported factors that improve prognosis include the absence of lymph node metastasis, the long period from the treatment of the primary lesion to the appearance of metastasis, and the slow progression of metastatic lesions [26–28].

**Table 1:** Reports of pancreatic resection for metastatic ocular malignant melanoma

Author	Gender	Age	Interval (year)	Diagnosis	Tumor size (cm)	Operative method	Additional treatment	Follow-up period (month)	Prognosis
Johansson et al. (1970) [21]	F	79	12	NA	NA	PD	None	11	Alive w/o rec Alive without recurrence
Camp et al. (2002) [22]	F	62	6	CT	5	DP + S	None	20	Alive without recurrence
Nifkarjam et al. (2003) [23]	F	45	12	CT MRI PET-CT	3	PPPD	None	6	Alive without recurrence
	M	55	13	CT PET-CT	NA	TP	None	7	Alive without recurrence
Vagefi et al. (2010) [19]	F	57	28	CT MRI PET-CT	2.2	DP + S	None	NA	NA
He et al. (2010) [20]	M	39	5	CT MRI	18	DP + S	Adjuvant chemotherapy	25	Alive without recurrence
De Maura et al. (2016)[24]	F	58	NA	CT EUS–FNA	4	PD	Adjuvant chemotherapy	NA	Alive without recurrence
Our case	F	48	5	CT EUS PET-CT	2.7	DP + S	Adjuvant chemotherapy	40	Alive with the recurrence

*M* male; *F* female; *NA* not available; *CT* computed tomography; *MRI* magnetic resonance imaging; *PET-CT* positron emission tomography-computed tomography; *EUS* endoscopic ultrasonography; *EUS-FNA* endoscopic ultrasound-guided fine-needle aspiration; *DP* distal pancreatectomy; *PD* pancreaticoduodenectomy; *S* splenectomy; *PPPD* pylorus-preserving pancreaticoduodenectomy; *TP* total pancreatectomy

In this case, the tumor was detected as a hypoechoic image mainly in the fourth layer of the gastric wall on preoperative EUS, and it was diagnosed as GIST because of the presence of blood flow inside. A leiomyoma or schwannoma can be differentiated as a clear mass with hypoechoic images mainly in the fourth layer of the gastric wall on EUS [29]. Endoscopic ultrasound-fine-needle aspiration (EUS-FNA) was necessary for a definitive diagnosis. However, since the attachment between the stomach and tumor was slight, and because color doppler showed blood flow in the mass, no puncture was performed considering the possibility of seeding and bleeding during EUS-FNA. The intraoperative discovery of a solitary pancreatic metastasis with no lymph node metastasis meant that radical resection could be performed, although multiple lung metastases and left renal metastases were found 4 months later. Nevertheless, with the continuation of chemotherapy, the patient survived at least 90 months after the initial treatment for malignant choroidal melanoma without any exacerbation of the tumor or any subjective symptoms.

Since malignant melanoma of the uvea spreads hematogenously [9], we thought that local resection without lymph node dissection would be sufficient. Furthermore, de Rooij et al. reported an improved quality of life following minimally invasive distal pancreatectomy, with no significant difference in operative blood loss or complication rates compared to open surgery for pancreatic tumor confined to the pancreas without vascular involvement [30]. Minimally invasive surgery is an appropriate option for oligo metastasis of the pancreas if complete resection of the tumor is possible.

The availability of molecular-targeted drugs for unresectable or recurrent melanoma has prolonged survival from a median of 10 months [31]. Several drugs are approved for malignant melanoma, including nivolumab (an antibody against PD-1), the proto-oncogene B-Raf inhibitor vemurafenib, ipilimumab (which targets CTLA-4), and the mitogen-activated extracellular kinase inhibitor trametinib [18]. Recent advances in immunotherapy and selective molecular targeted inhibitors have dramatically improved the outcomes of malignant melanoma [32]. Multiple studies have reported a median overall survival (OS) greater than 20 months after resection of uveal melanoma metastases [33]. In contrast, the OS was less than 13 months for checkpoint inhibitors and less than 12 months for targeted therapies, and these treatments were not superior to resection [34, 35]. For these reasons, the NCCN Guidelines for Melanoma: Uveal version 2. 2021 state that for resectable metastatic disease, the best outcomes are realized when complete resection is achieved [33]. At this time, complete resection is the first choice for patients with resectable tumors who can tolerate the procedure. In addition, dabrafenib and trametinib combination therapy and monotherapy with nivolumab or pembrolizumab have been approved for postoperative adjuvant chemotherapy, and improvement in therapeutic results after resection can be expected [18]. For an ectopic recurrence of malignant melanoma with a poor prognosis, complete resection should be the first-line treatment, followed by adjuvant chemotherapy, including molecular-targeted drugs and immune checkpoint inhibitors. In this case, a long-term prognosis was achieved by adding adjuvant chemotherapy after resection.

## Conclusion

Radical laparoscopic resection was possible for a solitary pancreatic metastasis originating from malignant choroidal melanoma. When coupled with adjuvant molecular-targeted chemotherapy for additional metastases, this approach leads to long-term survival.

## Data Availability

Not applicable.

## Abbreviations

PET: Positron emission tomography
CT: Computed tomography
EUS: Endoscopic ultrasonography
GIST: Gastrointestinal stromal tumor
EUS-FNA: Endoscopic ultrasound-fine-needle aspiration
FDG: Fluorodeoxyglucose
